# The effect of valgus reduction on resistant subtrochanteric femoral non-unions: a single-centre report of twenty six cases

**DOI:** 10.1007/s00264-023-06085-1

**Published:** 2024-01-16

**Authors:** Barakat El-Alfy, Alaa Abououf, Ahmed Darweash, Salam Fawzy

**Affiliations:** 1 Department of Orthopaedic Surgery, Mansoura University Hospital, Mansoura University, Mansoura, 7650001 Egypt; 2 Department of Orthopaedic Surgery, Faculty of Medicine, Aswan University, New Aswan City, 81528 Egypt; 3 Department of Orthopaedic Surgery, Faculty of Medicine, Suez University, PO Box 43221, Suez, Egypt

**Keywords:** Re-revision, Subtrochanteric, Non-union, Valgus, Contoured DCS

## Abstract

**Purpose:**

Re-revision of subtrochanteric non-unions is technically challenging and lacks robust evidence. The results of managing subtrochanteric fractures after multiple failed procedures have rarely been reported in the literature. This study aims to evaluate the effect of valgus reduction on non-united subtrochanteric fractures with single or multiple failed revision surgeries.

**Methods:**

Twenty-six patients with aseptic subtrochanteric fracture non-union underwent failed single or multiple revision procedures after index fracture fixation surgery between 2011 and 2019. The exclusion criteria were as follows: septic non-union, peri-prosthetic, and pathological fractures. Lateral-based wedge valgus reduction and compression at the non-union site using a valgus-contoured DCS together with decortication, debridement, and bone grafting were used. The main outcome measurement was radiological union, pain, LLD, HHS, and restoration of pre-fracture activities.

**Results:**

The mean follow-up was 4.5 years (range 3 to 7); prior revision surgeries range from two to five and union at 6.5 months (range 3 to 10) and the delayed union in one case and an infected non-union in one case. The mean LLD was 4 cm (range 3 to 5), which improved to 1.5 cm (range 1 to 4) (*P*-value < 0.001). The mean VAS was 7 (range 6 to 8), and 24 patients achieved painless ambulation without a walking aid after the union. The mean HHS was 40 (range 25 to 65), which improved to 85 (range 55 to 95) (*P*-value < 001), achieving 15 excellent, ten good, and one poor results.

**Conclusion:**

Mechanical optimisation by lateral closing wedge and stable fixation with pre-contoured DCS with biological enhancement resulted in a successful outcome in recalcitrant subtrochanteric non-unions.

## Introduction

Subtrochanteric fracture of the femur accounts for 10% to 34% of hip fractures and about 70% of proximal femur fractures [1–3]. Due to the subtrochanteric area’s anatomical and biomechanical features, the reported incidence of non-union, delayed union, and metal failure for any fixation device are up to 20% [4–6]. The subtrochanteric area is formed of thick cortical bone and extends at the metaphyseal–diaphyseal junction area with tenacious blood supply; the subtrochanteric area is the highest stressed zone in the human skeleton; the cantilever anatomy of the proximal femur creates an unequal distribution of stresses at proximal femur with medial cortex exposed to compression stress 20% more than the tensile stress acting on the lateral cortex, creating varus bending stress on any fixation device with impending failure if bone healing does not go in the expected timely manner [7–9]. Additionally, built-in anatomical and mechanical disadvantages, local tissue scarring, fibrosis, deformity, osteoporosis, bone defects created by osteosynthesis, and bone loss are more challenging factors increasing the difficulty of revision after index fracture fixation surgery[10, 11]. The treatment principles in those revisions are based on the diamond concept [12, 13] which gives the same importance to the mechanical and biological environment to achieve successful bone healing through debridement, decortication, bone grafting, and re-osteosynthesis with correction of varus malalignment [12–15]. In the case of multiple revision surgeries, the challenges are maximised, making a higher probability for recurrent non-union; so, the diamond concept could be ameliorated by valgus reduction in subtrochanteric non-unions; valgus reduction at the non-union site would shift the mechanical axis laterally, decreasing the lever arm and the tension force acting on the non-union site and converting them into a compression force. This will give a mechanical advantage that compensates for and optimises the corrected biological environment and stimulates bone healing.

Our study aimed to evaluate the effect of valgus reduction by fashioning a lateral closing wedge at the non-union site in a cohort of patients with resistant subtrochanteric non-union who underwent at least one failed revision surgery after the index fracture fixation surgery.

## Patients and methods

Between 2011 and 2019, the current prospective study was carried out at a single tertiary referral university medical facility. Twenty males and six females aged 22 to 67 years (mean age 45.56 years) were part of a cohort of 26 patients with aseptic subtrochanteric fracture non-union who had undergone failed single or multiple revision procedures after index fracture fixation surgery. Non-union was defined radiologically as the absence of bridging callus at three cortices at least six months following revision surgery, with or without evidence of implant failure [16, 17], and clinically as prolonged pain on weight-bearing 6 months after the last operation [18]. Exclusion criteria included failed revisions with septic non-union, peri-prosthetic, and pathological fractures (Table 1).

**Table 1:** Patient demographics

Male/female	20/6
^*^Age (years)	45.56 (22–67)
^**^Number of revisions	2 (1–3)
Failed implants withdrawn	
Dynamic condylar screw	10
Intramedullary nail	7
Proximal femur locking plate	9
^**^Time till union (weeks)	18 (12–40)
^*^Follow-up (months)	48 (36–84)
Complications:	
Delayed union	1 (3.8%)
Infected non-union	1 (3.8%)
Superficial wound infection	1 (3.8%)

Values are *n* (%) unless otherwise noted. ^*^Mean for normally distributed data and **median for non-normally distributed data

Preoperatively, septic non-union was ruled out by careful history taking, clinical examination, and serial results of inflammatory blood markers [19, 20].

**Fig. 1:**
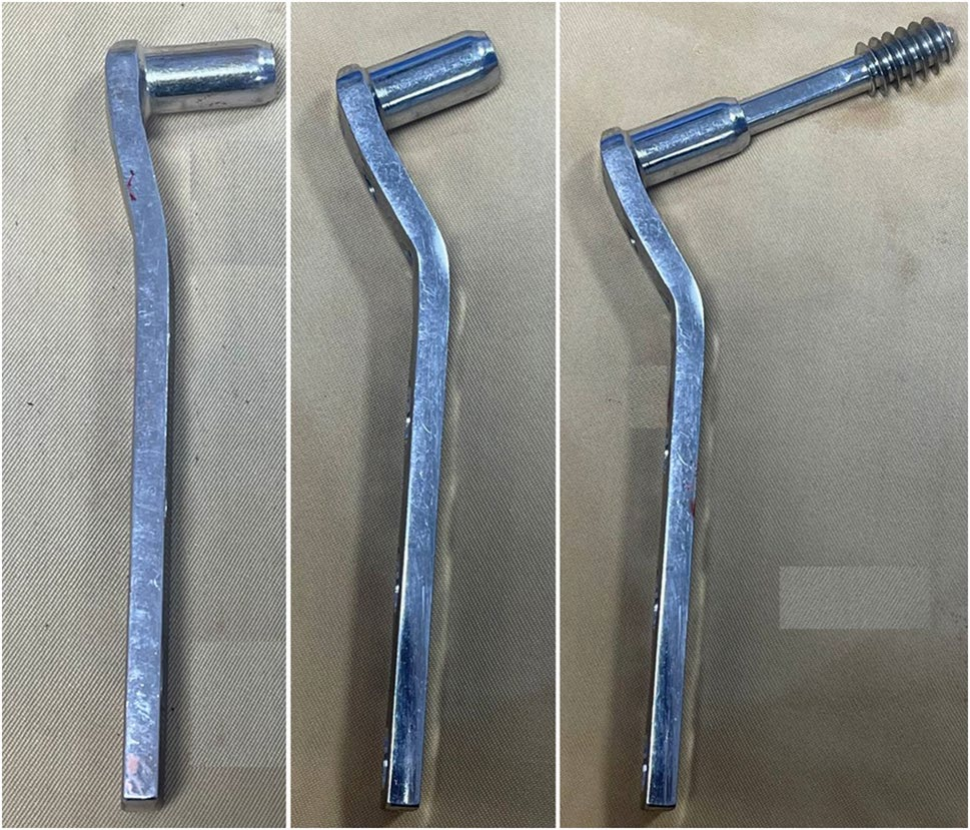
The DCS plate is contoured to fit for the valgus reduction and over-corrected neck-shaft angle

**Fig. 2:**
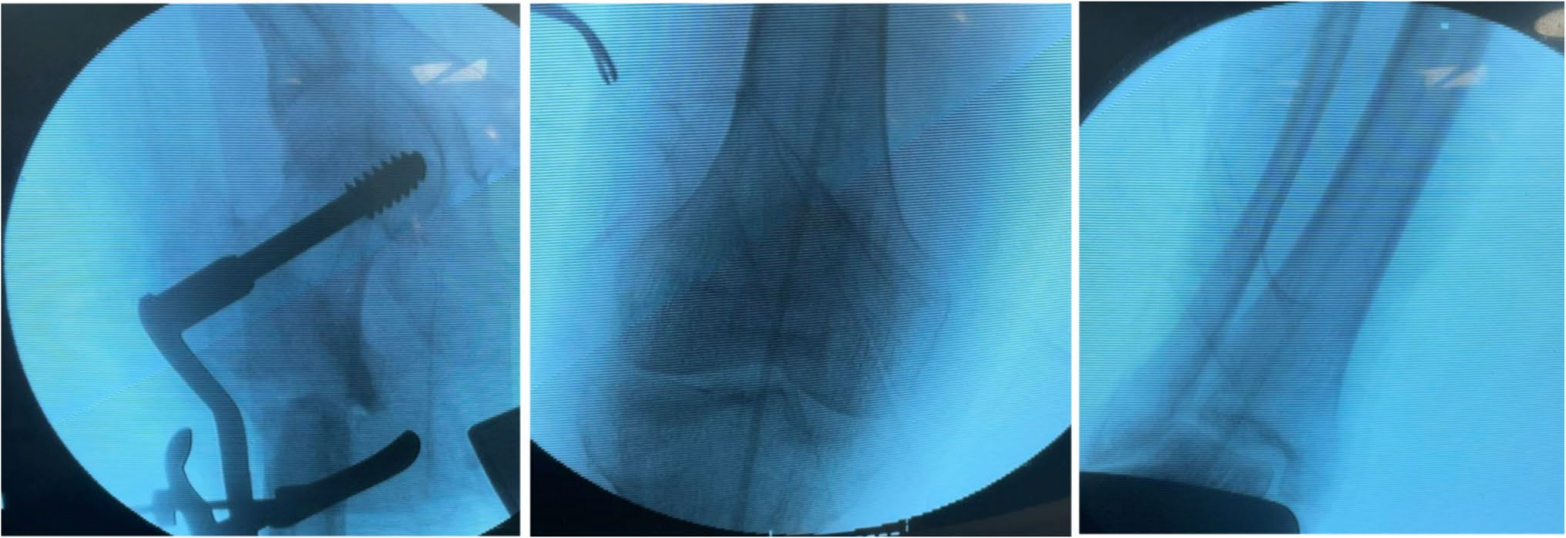
Preliminary fixation is done, and the mechanical axis is adjusted to pass just lateral to the medial cortex at the fracture site

All clearances, informed consents, and comorbidities were optimised; the hospital antibiotic policy administered a prophylactic antibiotic one h before surgery. After induction of anaesthesia, the patient was put supine on a traction table. The non-union site was reached using the previous surgical incision. The implant was removed; fibrous tissue excision and bone edges were refreshed at the fracture site. The bone ends were fashioned in a certain way using rongeurs, osteotomes, and rasping for fine toning of coapting surfaces to produce valgus when they were reduced as if we were doing a lateral closing wedge osteotomy. The lag screw is inserted under fluoroscopy control and used as a joystick to achieve reduction at the osteotomy site. The amount of valgus was adjusted to make the mechanical axis pass just lateral to the medial cortex at the non-union site. A malleable plate contouring template is moulded directly to the reduced lateral femoral cortex and used to contour the DCS plate to fit the created valgus at the non-union site and accommodate the over-corrected neck-shaft angle (Fig. 1). The side plate is applied, and the bone is reduced and fixed preliminary to the side plate. The mechanical axis is then rechecked by the diathermy cable extending from the centre of the hip to the centre of the ankle. It should pass lateral to the medial cortex at the non-union site. It will be shifted laterally in the knee but should not exceed the Fujisawa point (Fig. 2). Definitive fixation is then carried out. Screws on each side of the fracture site are inserted eccentrically to produce compression at the fracture site. The remaining screws could be locked or conventional, depending on the bone quality. The excised bonny parts are divided into small fragments and used as an impaction graft; however, an autogenous cancellous iliac bone graft was required in some cases. Muscle debridement wound closure in layers over the drain that was removed 48 h after surgery. The patients were discharged 72 h post-surgery after the last dose of antibiotic. Following surgery, the patient is allowed non-weight-bearing ambulation with crutches or a walker; a two week post-surgery follow-up is scheduled. Then, every six weeks, regular follow-ups are scheduled until the union and every six months interval after the union. Radiographic union was defined as bridging callus across three of four visible cortices on AP and lateral views and lacking implant slippage or breakage [11]. Weight-bearing progresses from partial (with early callus) to total when the callus spans at least three cortices. All patients were evaluated for pain, limp, limb-length discrepancy (LLD), hip and knee range of motion, radiological union, restoration of pre-fracture activities, and overall patient satisfaction.

### Statistical analysis

Descriptive statistical analyses were used; statistical analysis was performed using SPSS software version 25 for Windows (SPSS Inc., Chicago, IL, USA). Continuous data are presented as mean ± standard deviation (range) and categorical data as a percentage of patients. The normal distribution of data was tested using the Kolmogorov–Smirnov test. For normally distributed data, the paired *t*-test was used. The Wilcoxon signed-rank test was used to analyse non-normally distributed data. Statistical significance was indicated at *P*-value < 0.05.

## Results

**Fig. 3:**
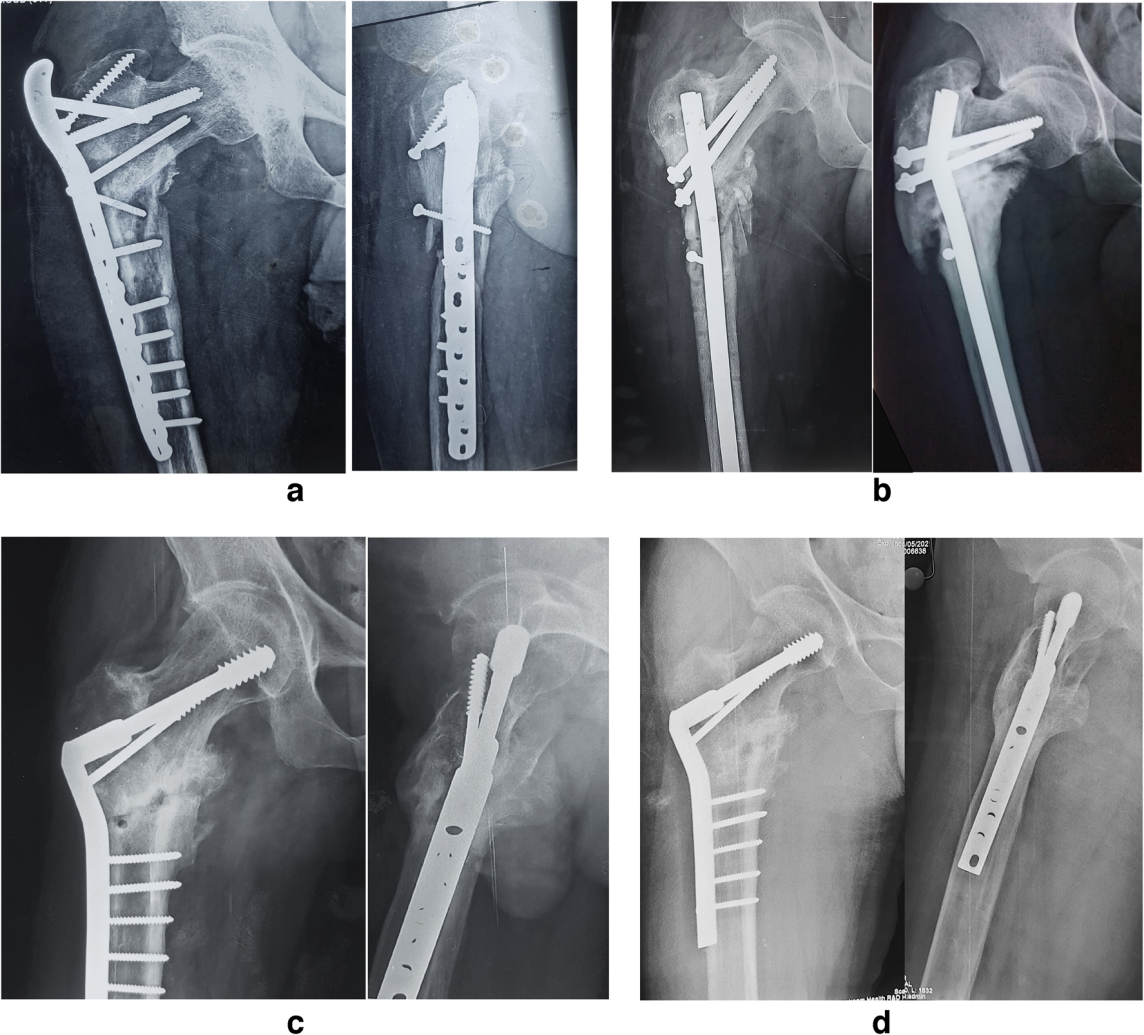
**a** A 32-year-old male patient with a subtrochanteric fracture fixed by a locked plate proximal femur, but he was complicated by varus collapse and non-union. **b** He was managed by proximal femoral nail and bone graft, but 5 months later, the nail was broken, and the fracture did not unite. **c** The nail was removed, and valgus reduction and fixation by contoured DCS were done. **d** Nine months postoperative with complete fracture healing

Twelve of the 26 patients had polytrauma-related fractures, although none of them had ipsilateral lower limb fractures. The mean number of prior revision surgeries was two revisions (range 1 to 3), and the mean follow-up was 48 months (range 36 to 84). Iliac autograft was needed in eight cases. The average valgus correction needed 10° (range 8 to 15°). All cases went into union except for one case; the mean time to union was 18 weeks (range 12 to 40). The delayed union in one case was treated with autogenous bone grafting and resolved by the tenth month after surgery. The other case experienced an infected non-union, and it was treated by debridement and with Ilizarov bone transport. Before surgery, the mean LLD was 3.54 cm (cm) (range 2 to 5), and it considerably improved to 2.26 cm (range 1 to 4) post-surgery (*P*-value < 0.001). A female patient with LLD, 4 cm after surgery, refused any lengthening procedures, was content with the shoe elevation and was satisfied with the procedure’s results. There were no intraoperative complications; one patient had a superficial infection treated with local treatment and systemic antibiotics without needing a second surgery. Before surgery, all individuals experienced considerable pain; the mean VAS was 7 (range 6 to 8), with ambulation utilising a walker, double crutches, or wheelchair bound. All patients achieved painless ambulation without a walking aid after union except one (*P*-value < 0.001). He was a 67-year-old male patient, the eldest in our cohort, who continued to use a single elbow crutch after the union. The pre-surgery mean Harris hip score (HHS) was 38 (range 18.5 to 51.5); the mean Harris hip score significantly improved to 89.62 (range 39 to 95) *P*-value < 0.001, achieving 15 excellent (57.7%), ten good (38.5%), and one poor result (3.8%) in three years of follow-ups as our intended endpoint for final evaluation (Table 2) (Fig. 3).

**Table 2:** Clinical outcome of surgery

	^**^VAS	^*^LLD	^*^HHS	*Excellent*	*Good*	*Fair*	*Poor*
Pre-operative	7	3.54	38	*0*	*0*	*0*	*26* *(100%)*
Post-operative	1	2.26	89.62	*15 (%57.7%)*	*10 (38.5%)*	*0*	*1 (3.8%)*
*P*-value	< 0.001	< 0.001	< 0.001				

*VAS* visual analogue scale, *LLD* limb-length discrepancy, *HHS* Harris hip score. ^*^Mean for normally distributed data and **median for non-normally distributed data. Statistical significance was indicated at *P*-value < 0.05

## Discussion

**Fig. 4:**
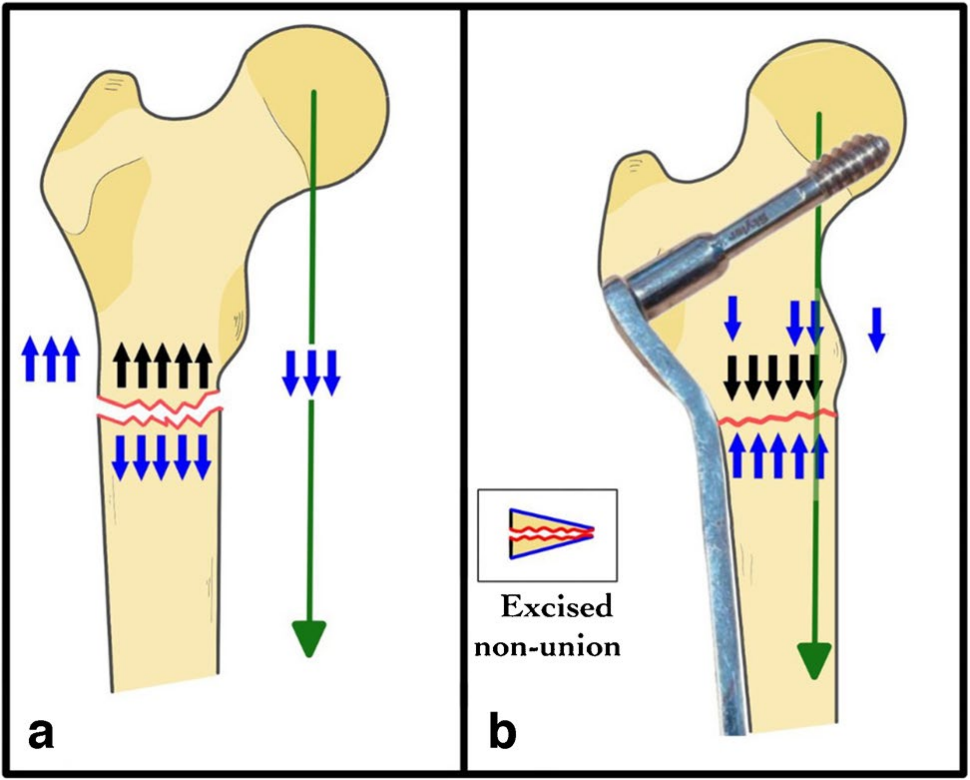
With a normal neck-shaft angle, the mechanical axis passes medial to the proximal femur, and this creates tension force at the subtrochanteric region lateral and bending stresses medially; both maximised with varus malalignment ending in failure (**a**) but in valgus reduction and side plate fixation; tensile stress converted into compression force, and the mechanical axis is shifted laterally and converts most of bending force into compression force (**b**)

Non-union of the subtrochanteric fracture causes pain and impairment of function. Patients with repeated failed revision procedures are typically debilitated, unsatisfied, and disappointed and have a seriously affected quality of life [11, 21]. Patients frequently appear with pain, limping, limited mobility, easy fatigability, varus collapse, LLD, and damaged implants after a botched revision surgery [22]. Many options are available for managing this difficult problem, but none has been proven ideal. The core idea in most of them is to improve the local biology, restore the normal alignment, and provide a stable fixation [11, 33]. However, this was insufficient to promote union in this area with exceptionally high stresses. The proximal femur is eccentrically loaded, and under physiological loads, the bending strains rise at the medial cortex, which increases the risk of subtrochanteric non-union and fixation failure [9, 23]. Adding a little valgus at the non-union site would improve the biomechanical environment and stimulate bone healing. In valgus reduction, the mechanical axis will be shifted laterally, reducing the lever arm of the tensile force acting on the non-union site and converting most of it into a compression force (Fig. 4). In this study, we used this concept to manage resistant subtrochanteric non-union in 26 cases. Our local bone debridement and decortication approach at the non-union site was fashioned into a lateral-based wedge with well-coapting sides. The degree of valgus angulation required to relocate the mechanical axis of the femur just lateral to the medial cortex of the femur determines the size of the wedge and contouring of the DCS side plate, ensuring the following: First, most of the bending forces are converted into compression forces via the well-reduced non-union site. The amount of valgus created at the non-union site improves the biomechanical environment and, at the same time, does not disturb the mechanics of the limb as the overall alignment remains within normal (Fig. 3). Second, the DCS side plate acts as a tension band plate, transferring tensile loads along the lateral side of the femur into compression stresses at the fracture site. In contrast to intramedullary nails, which have a tendency to varus mal reduction and are challenging to accomplish compression [23, 24], utilising an eccentric device can allow and maintain the valgus reduction. Third, it increases the healing surface area by neutralising the naturally narrow subtrochanteric area [11] and ensuring anatomical reduction of the medial cortex at the wedge’s apex, directly correlating with a lower risk of non-union. There is no need for lateral displacement at the osteotomy site because correcting osteotomy is achieved at the site of the Centre of Rotation of angulation (CORA), which negates the need for lateral displacement, unlike the case of subtrochanteric valgus osteotomy in cases of non-united neck of femur and intertrochanteric fracture, which requires obligatory lateral displacement at osteotomy site which is distal to site of CORA which is the non-union site [25]. Fourth, it improves limb-length discrepancy and mechanics of hip abductors by moving greater trochanter distally [26]. About 96% of patients in the present study achieved union with a significant improvement in pain and LLD. This may be attributed to the laterally based valgus wedge, which improves the power of hip abductors, which reduces pain fatigability and eliminates the need for walking. Limb length apparently improved secondary to correction of varus collapse, displacement, and malreduction at non-union site added to the bonus effect of valgus alignment. Several authors [11–13, 27] have used excess cortico-cancellous grafting of a subtrochanteric non-union revision surgery to improve local biology and healing. In our series, the bone chips from local decortication and wedge creation were used as a local graft in most patients, and an iliac bone graft was not required except in eight patients. At three year follow-ups, 25 out of 26 patients had nearly fully recovered their socially engaged lives, achieving good and excellent functional results and increased quality of life. There were no cases of increased hip pain or reduction in Harris hip score during the follow-up period, which extended in some patients up to seven years, with no evidence on follow-up x-rays of advancing hip osteoarthritis or avascular head necrosis that could be attributed to changes in hip reaction forces caused by subtrochanteric valgus osteotomy [26, 28]. Similar studies that describe the results of treating refractory subtrochanteric non-union take much work to come by using IMN, DCS, and angled blade plate fixation along with bone grafting. Haidukewych et al. [11] reported the results of 23 subtrochanteric non-union. Of the 23 patients, 12 had previously undergone revision; only five had undergone more than one revision. They reported overall success in 80% of the cases. Lotzien et al. [29] revised 40 patients with aseptic subtrochanteric non-union following failed IMN; they used DCS to correct the neck-shaft angle mean pre-operative 118° (range 101–131°) to 128° (range 114–142°), with bone grafting, DCS side plate, and anterior plating in 18 cases. Union was achieved in 67.5% of cases and metal failure and non-union in 25%, and a second operation was required in 32.5%. The average healing duration was 11.6 months; only 37.5% of cases required no walking aid. Although they attempted to resist failure risk factors, they aimed at normal restorative anatomy, which provided limited optimization of the mechanical environment, which could explain our results’ considerable improvement. The “diamond concept” has recently been used to refer to a conceptual framework for a successful bone repair response in cases of non-union, giving equal importance to mechanical stability and the biological environment, including both the multidimensional biological bone healing pathways (bone grafting) and the enhancement of the mechanical environment (fixation revision) [12, 13, 15]. Giannoudis et al. [27] performed revision surgery on 14 patients with subtrochanteric non-union using an angled blade plate fixation, all with bone graft composite obtained by reaming contralateral femur mixed with stem cells obtained by processing bone marrow aspiration from the iliac crest, and they achieved healing at an average of 6.8 months (range 5–12), with one case required re-revision due to metal failure. In a series of 12 patients with subtrochanteric non-union treated with local debridement and dual plating using an anterior plate added to the proximal femoral locked plate, plus excess cancellous bone grafting at the fracture site harvested from iliac bone, Mittal et al. [14] reported an average healing time of seven months (range 5 to 9). In our case series, a healing time of 18 weeks (range 12 to 40) was achieved, which is comparatively better when considering the debilitated and decompensated bone and soft tissues because of multiple surgeries. These effects were partially offset by the improved mechanics brought about by reshaping the non-union site, which also improved the local biology by the locally produced graft since we only required an iliac bone graft in eight cases. We expanded the diamond concept metaphorically by adapting variables that point in the direction of failure to point in the direction of success.

In this study, we converted the subtrochanteric non-union, known by its resistance to the union, into a subtrochanteric valgus osteotomy, known for its high healing potential. To the best of our knowledge, this concept has not used before in managing subtrochanteric non-union.

Our study’s strength, in addition to being a prospective study, is that it covers a high number of cases with extensive follow-up. The limitations are the lack of a control group, which can be attributed to the rarity of patients, and the lack of laboratory or cadaveric studies, as we based our work on plenty of evidence reported in the literature regarding the success of subtrochanteric valgisation osteotomy in managing femoral neck non-union.

## Conclusion

Recalcitrant subtrochanteric non-unions are more challenging to treat. Combining debridement, resection of the bone edges, valgus reduction, and fixation by contoured DCS plate with or without iliac bone graft resulted in a successful, encouraging outcome in managing such difficult cases. It is a valid option to consider when treating patients with aseptic subtrochanteric non-unions who have experienced single or multiple failed revision surgeries.

## Data Availability

Raw data is available for review.
